# Association between the serum uric acid/serum creatinine ratio and cognitive function in older adults: NHANES in the United States

**DOI:** 10.1038/s41598-024-67580-y

**Published:** 2024-07-15

**Authors:** Gang Chen, Ling Tong, Qing Ye

**Affiliations:** 1 Department of Laboratory Medicine, The Children’s Hospital, Zhejiang University School of Medicine, National Clinical Research Center for Child Health, National Children’s Regional Medical Center, Hangzhou, 310052 China; 2 Sir Run Run Shaw Hospital, Zhejiang University School of Medicine, Hangzhou, China

**Keywords:** Serum uric acid, Serum creatinine, SUA/SCR ratio, Cognitive impairment, Cognitive ageing, Cognitive neuroscience, Nephrology

## Abstract

Cognitive impairment can potentially become a significant health concern in older adults. However, early effective diagnostic methods are still lacking. Therefore, we utilized the NHANES database in the US to investigate the relationship between serum uric acid to serum creatinine (SUA/SCR) ratio and cognitive impairment. In our study, a total of 3874 participants were included (2001–2002, 2011–2014). Weighted t tests or chi-square tests were utilized to analyze the basic characteristics of the population. Weighted logistic regression analysis, smooth-fit curves, threshold effects, and subgroup analysis were conducted to investigate the correlation between the SUA/SCR and cognitive impairment. In this study, the SUA/SCR was significantly lower in individuals with cognitive impairment. The logistic regression model, after adjusting for all covariates, revealed that the Q2–Q4 were 0.65 (95% CI 0.49, 0.86), 0.60 (95% CI 0.40, 0.90), 0.55 (95% CI 0.39, 0.77) respectively. This indicates that participants in the Q4 had a 45% reduced risk of cognitive impairment. Smooth-fit curves and threshold effect analysis revealed a nonlinear relationship between SUA/SCR and cognitive impairment, with a turning point at 4.13. Subgroup analysis showed no statistically significant differences in the relationship between SUA/SCR and cognitive impairment among different subgroups (*P* > 0.05). Our findings indicate a negative correlation between the SUA/SCR and the risk of cognitive impairment in the population of adults aged 60 and above in the US. This suggests that the SUA/SCR holds promise as a potential indicator for cognitive impairment.

## Introduction

With increasing global life expectancy, cognitive impairment has the potential to become a major health challenge for older adults. However, without preventive measures to delay cognitive impairment, the progression of the condition will worsen, eventually leading to the development of Alzheimer’s disease (AD)^1^. It has been reported that in the United States alone, there were approximately 6.2 million AD patients in 2021, and it is projected to reach 13.8 million by 2026^2^. As the progression from cognitive impairment to AD is continuous and irreversible, and there are currently no effective treatments available^3^, it is crucial to identify and mitigate modifiable factors as early as possible.

Serum uric acid (SUA) is the end product of purine and uric acid(UA) metabolism^4^, serving as both an indicator of renal function and an effective antioxidant in the human body^5,6^. SUA levels depend on the balance between dietary purine intake, xanthine oxidase activity, and renal excretion of UA^7^. Disruption of this balance can lead to hyperuricemia or hypouricemia. Research has indicated that SUA may have a protective effect on cognitive impairment^8^. Serum creatinine (SCR) is a byproduct of muscle metabolism and a common biomarker used to assess renal function^9^. However, 90% of serum uric acid is filtered and reabsorbed by the kidneys^10,11^. Therefore, it is necessary to consider the impact of renal function when assessing the correlation between SUA and cognitive impairment. Recent studies have shown that the serum uric acid/serum creatinine ratio (SUA/SCR) as a novel biomarker believed to better reflect endogenous UA levels^12^. It has been reported that the SUA/SCR ratio is associated with the progression and prognosis of chronic kidney disease, metabolic syndrome, and respiratory system diseases^13–15^. However, the relationship between the SUA/SCR ratio and cognitive impairment has not been widely explored.

Common methods for assessing cognitive function include psychological tests, questionnaires, imaging examinations, and cerebrospinal fluid analysis^16,17^. Compared to other testing methods, serum biomarkers offer advantages in terms of objectivity and accessibility, making them more suitable for the early identification of cognitive impairment. Similarly, there is a lack of targeted early interventions for cognitive impairment. Therefore, based on the current situation, this study aims to select participants aged 60 and above from the National Health and Nutrition Examination Survey (NHANES) to investigate the potential association between SUA/SCR and cognitive impairment.

## Method

### Data sources and study population

NHANES is a research program to assess the health and nutritional status of adults and children in America. This study utilized data from the 2001–2002 and 2011–2014 NHANES cycles for analysis. Prior to data collection, all research procedures were authorized by the Ethics Review Board of the National Center for Health Statistics. Informed consent was obtained from all participants, and the study followed the guiding principles of the Declaration of Helsinki.

**Figure 1:**
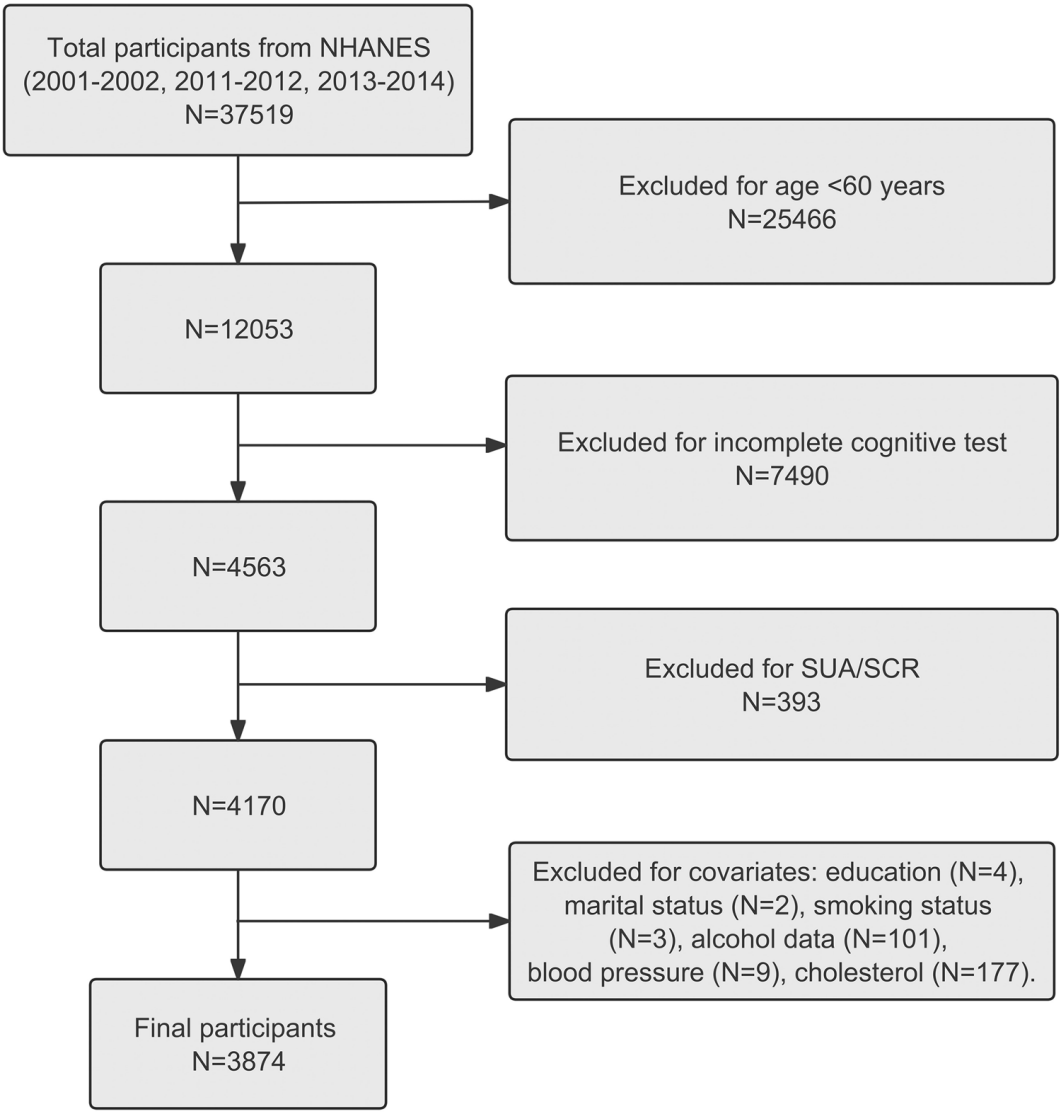
Screening flowchart.

In the 2001–2002 and 2011–2014 NHANES data, a total of 37,519 participants were included. Participants under the age of 60, those lacking data on the Digit Symbol Substitution Test (DSST), SUA/SCR, education level, marital status, smoking, alcohol consumption, blood pressure, and cholesterol were excluded. Finally, our study included data from 3874 participants Fig. 1.

### Study variables

#### Cognitive impairment

The DSST is a test that requires participants to accurately encode a series of symbols within 120 s. This test assesses the participants’ reaction speed, attention, spatial perception, associative learning, and memory, making it a sensitive indicator of cognitive function^18^. As there is no established DSST score threshold to define cognitive impairment, we defined cognitive impairment (DSST < 33) using the lowest unweighted quartile of DSST scores among the study participants, consistent with previous research methods^19^.

#### SUA/SCR

The SCR concentration was measured using the timed endpoint method with the DxC800. The DxC800 utilizes the Jaffe rate method (kinetic alkaline picrate) in the modular chemistry section to determine the concentration of SUA.

### Covariates

The variables included age, gender, race, marital status, BMI, education level, hypertension, hyperglycemia, hypercholesterolemia, alcohol consumption, and smoking status. Race was categorized as Mexican American, non-Hispanic White, non-Hispanic Black, other Hispanic, and other race. Marital status was divided into married/cohabiting, widowed/divorced/separated, and never married. BMI was categorized as < 25 kg/m^2^, 25–30 kg/m^2^, and > 30 kg/m^2^. Alcohol consumption was defined as consuming at least 12 alcoholic beverages per year, and smoking is defined as smoking at least 100 cigarettes over a lifetime^20^. Hypertension and hypercholesterolemia were defined as being informed by a healthcare professional, while diabetes was defined as being informed by a healthcare professional or having a glycated hemoglobin (HbA1c) level ≥ 6.5 mmol/L or fasting blood glucose ≥ 7.0 mmol/L.

### Statistical analysis

We selected “wtmec2yr” as the weighting variable for the years 2001–2002 and 2011–2014. This is consistent with previous research methods^21^. We conducted weighted t tests or chi-square tests to analyze the data of continuous and categorical variables for the basic characteristics. Continuous variables are presented as the mean ± standard error, while categorical variables are presented as 95% confidence intervals. To assess the association between SUA/SCR and cognitive impairment, we employed weighted logistic regression analysis. In Non-adjusted Model, no adjustment for confounding factors was made. In Partially adjusted model, we included adjustments for age and race. In Full adjusted model, we included age, gender, race, education, marital status, smoking status, alcohol consumption, BMI, hypertension, hypercholesterolemia, and diabetes for adjustment. Furthermore, after adjusting for all covariates, we employed a generalized additive model (GAM) and smooth curve fitting to observe non-linear correlations. In the presence of non-linear correlations, a segmented regression model was used to estimate threshold effects. The recursive experimental approach was utilized to identify the best-fitting model with the maximum likelihood value and determine the inflection point^22^. Finally, we performed subgroup analysis using a stratified multivariable logistic regression model to assess the correlation between SUA/SCR and cognitive function. The stratification variables included gender, age, BMI, smoking status, alcohol consumption, hypertension, hypercholesterolemia, and diabetes. These stratification variables were also considered potential effect modifiers. The interaction terms were evaluated using likelihood ratio tests to assess their heterogeneity. All data were analyzed using R (version 4.2.0) and SPSS (version 27).

### Institutional review board statement

The NHANES survey protocol was approved by the CDC’s National Center for Health Statistics Institutional Research Ethics Review Board.

## Results

### Baseline characteristics of the study population

A total of 3874 individuals participated in this study, with 1900 males and 1974 females. The study population was divided into two groups based on the lowest quartile score of DSST: individuals with DSST < 33 were classified as having cognitive impairment, while those with DSST ≥ 33 were classified as having noncognitive impairment. Noncognitive impairment was more prevalent among the Non-Hispanic White population, individuals with higher education levels, and those without hypertension. On the other hand, cognitive impairment was more commonly observed among females, older individuals, and those who reported alcohol consumption (*P* < 0.05). Interestingly, the SUA/SCR ratio was significantly lower in the cognitive impairment group (*P* < 0.05). Table 1

**Table 1:** Weighted characteristics of the study population based on cognitive impairment.

	Non-Cognitive impairment	Cognitive impairment	*P*-value
Sex (%)			0.436
Male (n = 1900)	45.37 (43.43, 47.32)	47.25 (43.22, 51.32)	
Female (n = 1974)	54.63 (52.68, 56.57)	52.75 (48.68, 56.78)	
Age (mean ± se)	68.82 ± 0.16	73.37 ± 0.35	< 0.001
Age, (%), year			< 0.001
60–69	58.59 (56.36, 60.78)	31.36 (27.11, 35.95)	
70–79	30.05 (28.07, 32.10)	36.22 (32.18, 40.47)	
≥ 80	11.37 (10.15, 12.72)	32.42 (27.57, 37.67)	
BMI (mean ± se)	28.85 ± 0.19	28.80 ± 031	0.899
BMI (%), kg/m^2^			0.510
< 25	26.19 (23.64, 28.91)	26.49 (22.35, 31.09)	
25–30	38.75 (36.61, 40.93)	41.30 (36.84, 45.91)	
> 30	35.06 (32.74, 37.46)	32.21 (27.43, 37.39)	
Race (%)			< 0.001
Mexican american	2.33 (1.64, 3.30)	8.24 (5.51, 12.15)	
Other hispanic	2.17 (1.51, 3.10)	11.74 (8.08, 16.75)	
Non-Hispanic white	84.98 (82.32, 87.31)	58.07 (52.00, 63.91)	
Non-Hispanic black	5.97 (4.65, 7.65)	18.06 (14.42, 22.36)	
Other race	4.54 (3.47, 5.94)	3.90 (2.30, 6.54)	
Education (%)			< 0.001
Less than 9th grade	3.09 (2.35, 4.06)	29.42 (25.42, 33.76)	
9–11th grade	9.40 (7.96, 11.06)	22.90 (18.92, 27.43)	
High school graduate	23.38 (20.93, 26.02)	24.08 (20.46, 28.11)	
Some college or AA degree	31.62 (28.79, 34.59)	16.38 (12.91, 20.57)	
College graduate or above	32.51 (29.08, 36.14)	7.22 (5.12, 10.09)	
Marital status (%)			< 0.001
Married/Living with partner	67.68 (65.68, 69.62)	47.72 (42.60, 52.89)	
Widowed/divorced/separated	28.64 (26.89, 30.47)	46.13 (40.80, 51.56)	
Never married	3.67 (2.85, 4.72)	6.15 (4.05, 9.23)	
Smoking status (%)			0.857
Yes	50.79 (47.82, 53.75)	51.30 (46.60, 55.98)	
No	49.21 (46.25, 52.18)	48.70 (44.02, 53.40)	
Alcohol consumption (%)			< 0.001
Yes	72.32 (69.26, 75.18)	56.68 (53.08, 60.22)	
No	27.68 (24.82, 30.74)	43.32 (39.78, 46.92)	
Hypertension (%)			< 0.001
Yes	55.53 (53.02, 58.01)	69.15 (64.45, 73.49)	
No	44.47 (41.99, 46.98)	30.85 (26.51, 35.55)	
Hypercholesterolemia (%)			0.819
Yes	55.74 (53.18, 58.28)	55.15 (50.05, 60.15)	
No	44.26 (41.72, 46.82)	44.85 (39.85, 49.95)	
Diabetes (%)			0.084
Yes	39.51 (36.76, 42.34)	34.49 (30.24, 39.00)	
No	60.49 (57.66, 63.24)	65.51 (61.00, 69.76)	
SUA/SCR (mean ± se)	6.10 ± 0.04	5.66 ± 0.06	< 0.001

Continuous variables were expressed as mean ± se, while categorical variables were expressed as 95% CI. BMI stands for body mass index, and SUA/SCR represents the ratio of serum uric acid to serum creatinine. *P* < 0.05 was considered statistically significant.

### Association between SUA/SCR and cognitive impairment

To further investigate the relationship between SUA/SCR and cognitive function, we employed a weighted logistic regression model to analyze their association. When adjusting for covariates, the fourth quartile of SUA/SCR showed a 43% reduction in cognitive impairment risk compared to the first quartile (OR 0.57; 95% CI 0.46, 0.71). After adjusting for some covariates, age, and race, we observed the same trend. After adjusting for all covariates, Q1, Q2, Q3, and Q4 were 1.00, 0.65 (0.49, 0.86), 0.60 (0.40, 0.90), and 0.55 (0.39, 0.77), respectively. Compared to the first quartile, participants in the fourth quartile had a 45% reduced risk of cognitive impairment (OR 0.55; 95% CI 0.39, 0.77), *P* for trend = 0.003. Table 2

**Table 2:** The correlation between SUA/SCR and cognitive impairment was analyzed using weighted logistic regression.

Categories	Non-adjusted model	Partially adjusted model	Full adjusted model
Quartile 1	1 (Ref)	1 (Ref)	1 (Ref)
Quartile 2	0.72 (0.58, 0.91) 0.007	0.68 (0.54, 0.86) 0.002	0.65 (0.49, 0.86) 0.004
Quartile 3	0.60 (0.45, 0.79) < 0.001	0.63 (0.45, 0.86) 0.006	0.60 (0.40, 0.90) 0.016
Quartile 4	0.57 (0.46, 0.71) < 0.001	0.62 (0.49, 0.80) < 0.001	0.55 (0.39, 0.77) 0.001
*P* for trend	< 0.001	0.005	0.003

Non-adjusted model: No adjustment for covariates was made. Partially adjusted model: Adjustment was made for age and race. Fully adjusted model: Adjustment was made for age, gender, race, education, marital status, smoking status, alcohol consumption, BMI, hypertension, hypercholesterolemia, and diabetes. Data are presented as OR (95% CI), *P*-valued. 95% CI, 95% confidence interval. OR, Odds Ratio. *P* < 0.05 was considered statistically significant.

### Using a generalized additive model, we explored whether there was a linear relationship between SUA/SCR and cognitive impairment

**Figure 2:**
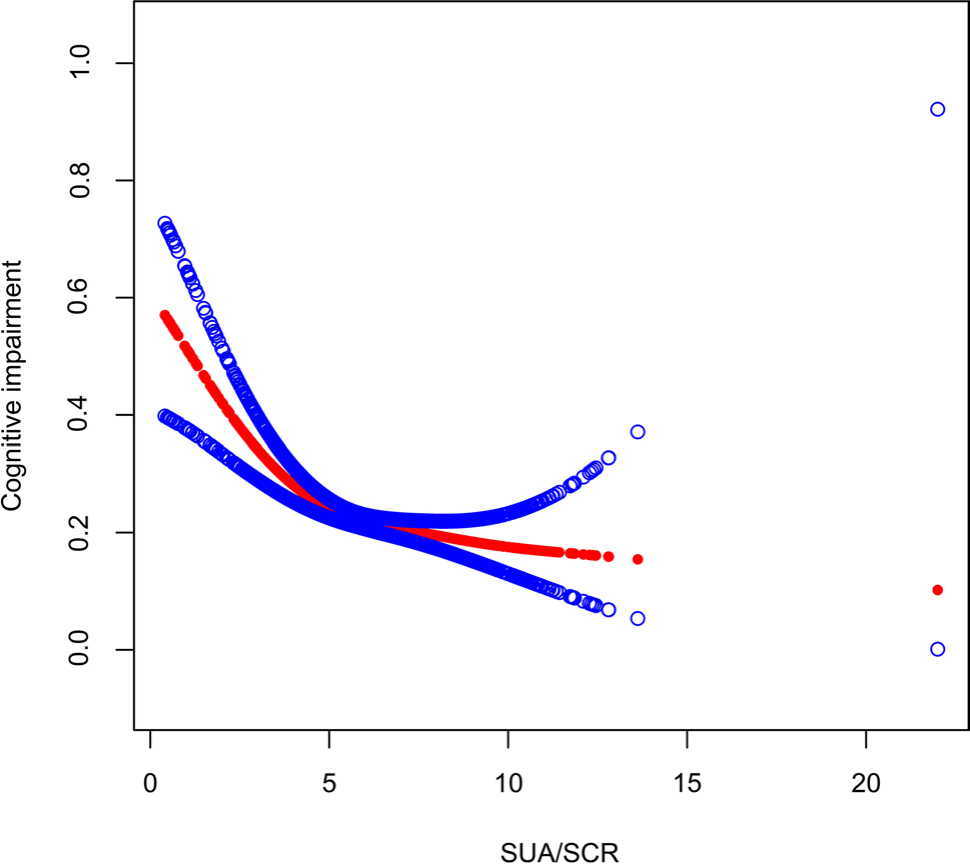
The association between SUA/SCR and cognitive impairment. The red line represents the smoothed curve fit between the variables, while the blue line represents the fitted 95% confidence interval.

To validate the reliability and stability of the logistic regression analysis results, we conducted an analysis using a generalized additive model while adjusting for all covariates. We observed a nonlinear association between SUA/SCR and cognitive impairment. Furthermore, through threshold effects, we identified a turning point at 4.13. On the left side of the turning point, the effect size was 0.611 (95% CI 0.487, 0.767), and on the right side of the turning point, the effect size was 0.930 (95% CI 0.868, 0.997). A log likelihood ratio < 0.001. The above results indicated that a nonlinear relationship between SUA/SCR and cognitive impairment Fig. 2 and Table 3

**Table 3:** Threshold effect of SUA/SCR on cognitive impairment.

SUA/SCR	Adjusted OR (95% CI), *P* value
inflection point	4.13
SUA/SCR < 4.13	0.611 (0.487, 0.767) < 0.0001
SUA/SCR ≥ 4.13	0.930 (0.868, 0.997) 0.0407
Log likelihood ratio	< 0.001

Adjusted for age, gender, race, education, marital status, smoking status, alcohol consumption, BMI, hypertension, hypercholesterolemia, and diabetes. 95% CI, 95% confidence interval. OR, Odds Ratio. *P* < 0.05 was considered statistically significant.

### Subgroup analysis results of the association between SUA/SCR and cognitive impairment

**Figure 3:**
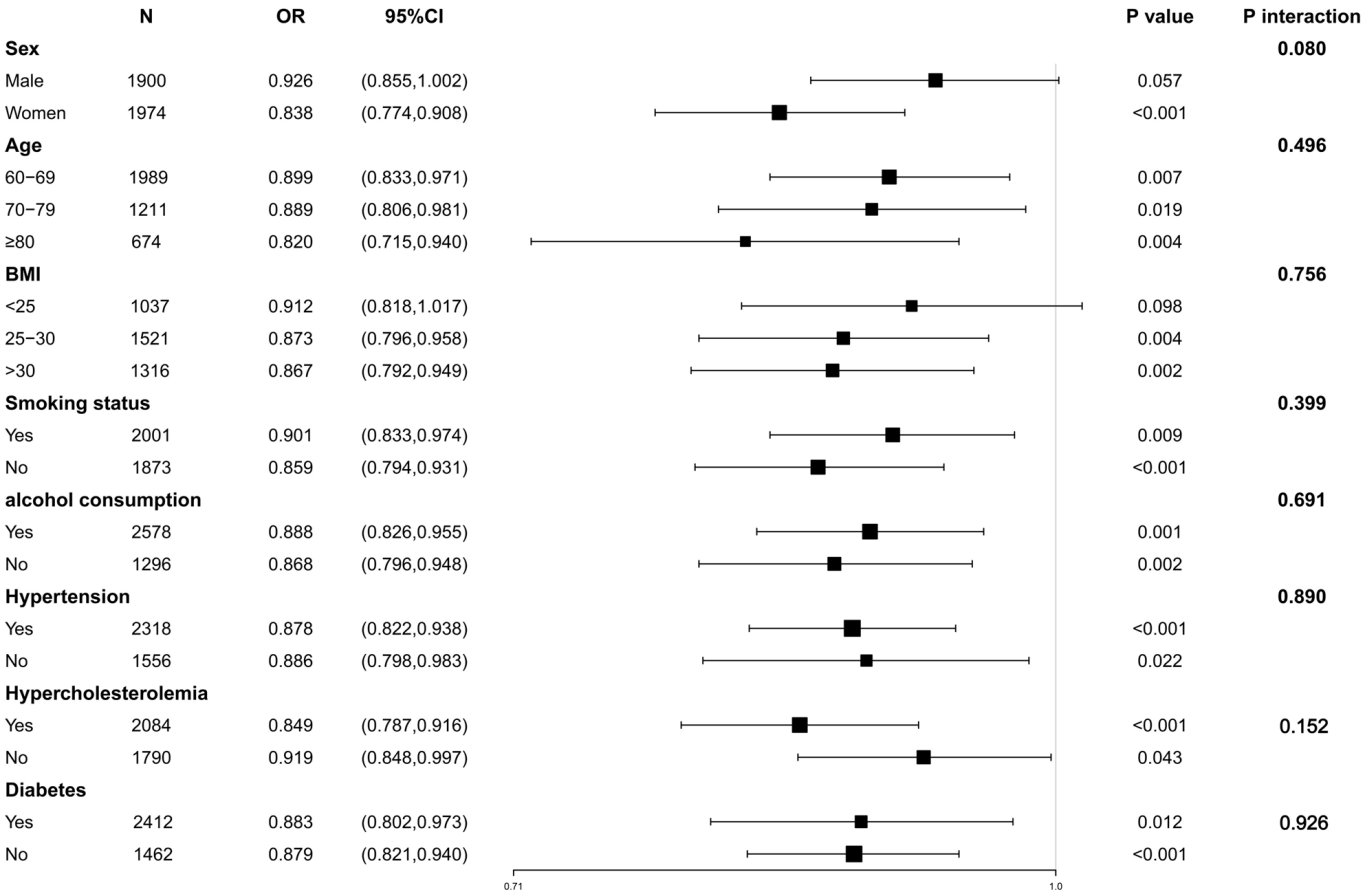
Subgroup analysis of the association between SUA/SCR and cognitive impairment.

Further subgroup analysis revealed that SUA/SCR was significantly associated with cognitive impairment (*P* < 0.05) in the subgroups stratified by age, smoking status, drinking status, hypertension, hypercholesterolemia, and diabetes. The correlation between SUA/SCR and cognitive impairment in the population with BMI < 25 and in the male population was not statistically significant (*P* > 0.05). This lack of significance may be attributed to the relatively small proportion of individuals in these two categories. The interaction analysis indicated no statistically significant differences in the relationship between SUA/SCR and cognitive impairment among different subgroups, suggesting that gender, age, BMI, smoking status, drinking status, hypertension, hypercholesterolemia, and diabetes did not significantly affect this negative correlation (*P* > 0.05) Fig. 3.

## Discussion

To the best of our knowledge, our study is the first investigation of the relationship between SUA/SCR and cognitive impairment among older adults in NHANES dataset. In this cross-sectional study, after adjusting for demographic, examination, and personal data of participants from the NHANES database in 2001–2002 and 2011–2014, we found a significant increase in the risk of cognitive impairment associated with a decrease in the SUA/SCR ratio. Additionally, through smooth curve fitting, we discovered a nonlinear relationship between SUA/SCR and cognitive impairment. The threshold effects analysis revealed a turning point at 4.13, and when SUA/SCR was below or above 4.13, both were independent risk factors for cognitive impairment. The subgroup analysis results indicated that, except for the subgroup of BMI < 25 and males, the association between SUA/SCR and cognitive impairment was statistically significant in the remaining subgroups. The lack of significance in these two subgroups may be attributed to the relatively small proportion of individuals. The results of the interaction analysis suggest that this association is consistent across different populations. Our research findings suggest that SUA/SCR exhibits a certain degree of stability in patients with cognitive impairment. In the future, SUA/SCR may serve as an effective indicator for assessing cognitive impairment.

Cognitive impairment initially manifests as memory loss, decreased executive function, language deficits, or impairments in visual-spatial abilities^23,24^. The specific mechanisms underlying cognitive impairment remain incompletely understood, and effective treatments are currently lacking^25,26^. Furthermore, the occurrence of cognitive impairment greatly diminishes the quality of life for individuals affected, and without effective intervention, it poses an increasing economic burden on nations, families, and individuals^27^. Therefore, early detection and intervention to modify contributing factors are of paramount importance.

As the end product of purine metabolism, UA maintains a steady balance through the combined effects of endogenous production, exogenous supply, excretion, and reabsorption. In recent years, many studies have focused on the relationship between uric acid and the development and prognosis of cognitive dysfunction. However, whether UA is a protective or risk factor for cognitive function remains controversial. A meta-analysis in 2023 demonstrated no significant association between SUA levels and the risk of cognitive impairment^28^. In contrast, some studies support the protective role of SUA against cognitive impairment. Barbara Iazzolino et al. found that high SUA significantly prevented cognitive impairment in amyotrophic lateral sclerosis^29^. A retrospective study emphasized the neuroprotective effect of high UA levels (> 237 mmol/L), possibly attributed to uric acid’s antioxidant properties^30^. Zelin Yuan et al. cross-sectional study on 6509 participants revealed an association between lower SUA and poorer cognitive function in females^31^. A prospective study found that low plasma UA levels had potential harmful effects on cognition^32^. Since 90% of UA in the serum is filtered and reabsorbed by the kidneys, UA levels in the blood are strongly influenced by renal function. Studies have shown that the effect of UA on cognitive impairment depends on renal function^10,11^. Therefore, assessing the relationship between SUA/SCR and cognitive impairment necessitates considering the influence of renal function. Research has found that SUA/SCR serves as a high-quality biomarker reflecting endogenous UA levels^33,34^.

In this study, we found that individuals with lower SUA/SCR ratios had a higher risk of cognitive impairment. In a recent prospective study involving 508 patients with Parkinson’s disease (PD), an investigation and analysis of cognitive impairment in PD patients revealed that a decrease in the UA to creatinine ratio was associated with increased accumulation of total-tau and phosphorylated-tau in cerebrospinal fluid. The study concluded that a decrease in the UA to creatinine ratio predicts the worsening of cognitive function in PD patients over time^35^. This finding is consistent with our research results. In our study, we observed a nonlinear negative correlation between SUA/SCR and the risk of cognitive impairment, where lower SUA/SCR ratios were associated with an increased risk of higher cognitive impairment. Additionally, the threshold effects analysis showed significant statistical significance on both sides of the turning point. Similarly, in our interaction analysis, we found that the relationship between SUA/SCR and cognitive impairment was not significantly influenced by the covariates we included. This suggests that SUA/SCR may have promising prospects in predicting cognitive impairment.

The exact mechanism by which the SUA/SCR ratio is involved in the development and prognosis of cognitive impairment remains unclear. Research has found that UA, an antioxidant, is produced during hypoxia^36,37^. However, oxidative stress in neurons is one of the main mechanisms influencing cognitive impairment^38^. Preclinical studies have shown that UA, as a natural antioxidant, can alleviate behavioral and cognitive impairments in MPTP-induced PD mice by activating the Nrf2-ARE pathway, thereby acting as a neuroprotectant by regulating neuroinflammation and oxidative stress^39^. Therefore, we speculate that the antioxidant action of SUA may be one of the protective mechanisms protecting against cognitive impairment, and lower uric acid levels may make patients more susceptible to cognitive impairment when renal function is similar.

Our study has several advantages compared to previous articles. First, we had a large sample size and applied weighted data analysis. Second, the smoothed fitted curves constructed based on a fully adjusted model helped us identify potential linear relationships. Finally, considering the possible impact of covariates on the results, our subgroup analysis may help to verify the stability of the results. However, our study also has certain limitations. For instance, the NHANES database is a cross-sectional survey, so we can only analyze the correlation between the SUA/SCR and cognitive impairment, and cannot investigate the causal relationship among them. Therefore, prospective studies are needed to verify these causal relationships, which will be a focus of our future research. Additionally, despite our efforts to include as many covariates as possible, we cannot entirely eliminate the interference of other potential confounding factors.

## Conclusion

In conclusion, our study findings suggest a negative correlation between the SUA/SCR ratio and the risk of cognitive impairment among older adults aged 60 and above in the United States. Further prospective studies are needed in the future to verify the potential mechanisms underlying this relationship.

## Informed consent

All participants provided written informed consent prior to enrollment.

## Data Availability

The datasets generated during the current study are freely available without restriction in the NHANES repository. (http://www.cdc.gov/nchs/nhanes.htm).
